# Trends of late HIV presentation and advance HIV disease among newly diagnosed HIV cases in Jiangsu, China: A serial cross-sectional study from 2008 to 2020

**DOI:** 10.3389/fpubh.2022.1054765

**Published:** 2022-12-08

**Authors:** Lingen Shi, Weiming Tang, Xiaoyan Liu, Haiyang Hu, Tao Qiu, Yuheng Chen, Xiaoqin Xu, Yunting Chen, Zhi Zhang, Ying Zhou, Jing Lu, Gengfeng Fu

**Affiliations:** ^1^Institute for STI and HIV Control and Prevention, Jiangsu Provincial Center for Disease Control and Prevention, Nanjing, Jiangsu, China; ^2^Jiangsu Key Laboratory of Molecular Medicine, Medical School, Nanjing University, Nanjing, Jiangsu, China; ^3^Project-China, University of North Carolina, Guangzhou, China

**Keywords:** HIV, late presentation, advanced HIV disease, associated factors, China

## Abstract

**Background:**

This study aimed to assess the trends and determine the factors associated with late presentation (LP) and advanced HIV disease (AHD) among newly diagnosed people living with HIV (PLWH) from 2008 to 2020 in Jiangsu, China.

**Methods:**

Newly diagnosed PLWH registered in the HIV surveillance system from 2008 to 2020 were included. Multivariable logistic regression models were used to analyze the factors associated with LP and AHD. The LP and AHD trends were assessed using Joint-point analysis.

**Results:**

Of 37,251 newly diagnosed PLWH identified, 30,251(81.2%) patients met the inclusion criteria. Among those, 16,672 (55.1%) were considered LP, and 8,691 (28.7%) had AHD. LP trends steadily increased from 2008 (39.0%) to 2020 (59.4%), but AHD trends decreased visibly from 2016 (32.3%) to 2020 (23.4%). The overall median CD4 trends decreased slowly from 389 to 305 cells/mm^3^ between 2008 and 2020. Married patients and those older than 35 years were more likely to be LP and have AHD. Patients infected via heterosexual transmission had a higher risk of being classified as AHD (aOR: 1.13, 95%CI: 1.06–1.21) than patients infected via homosexual transmission. Patients that were diagnosed at sexually transmitted infections (STIs) clinics (aOR: 1.10, 95%CI: 1.01–1.20) and in hospitals (aOR: 1.69, 95%CI: 1.59–1.79) were more likely to be classified as LP compared with patients diagnosed at voluntary counseling and testing (VCT) centers. Similar, patients diagnosed at STIs clinics (aOR: 1.23, 95%CI: 1.11–1.36) and hospitals (aOR: 2.27, 95%CI: 2.12–2.43) were more likely to have AHD than patients diagnosed in VCT.

**Conclusion:**

Our findings indicate an alarming burden of LP in Jiangsu, suggesting the need for more attention toward HIV diagnosis at early CD4 stages. National HIV control programs must strengthen comprehensive interventions for HIV prevention and promote HIV services. Also, strategies for HIV prevention (PrEP and PEP), testing, and treatment must be extended, especially among the general population.

## Introduction

Timely linkage to the care and antiretroviral therapy (ART) initiation plays multiple beneficial roles in HIV control and prevention (1). The timely diagnosis of an HIV infection is the first and crucial step to achieving benefits like preventing onward transmission and reducing the risks of acquired immune deficiency syndrome (AIDS) through early treatment initiation. Late presentation (LP) is a valuable marker that reflects the attendance of patients seeking HIV-related care early (2). LP could cause many consequences, such as increasing HIV related morbidity and mortality (3–7), high risks of onward HIV transmission (8–10), and cost of care (10). Advanced HIV disease (AHD) meant a lower CD4^+^ cell count when PLWH sought HIV related health care, which reduced their probability of survival even though they initiated ART immediately (11, 12). Meanwhile, AHD increases the odds of severe HIV-related clinical events like tuberculosis (13), severe bacterial infections, Cryptococcal meningitis, and toxoplasmosis (14). Therefore, monitoring trends in LP/AHD could identify HIV-related healthcare early and prevent further transmission.

Additionally, LP is a global issue of great concern to researchers as it hinders the achievement of the UNAIDS 95-95-95 goals toward ending HIV by 2030 (15, 16). Many studies have investigated the prevalence of LP and AHD using cross-sectional studies. One large cross-sectional study in Metropolitan France in 2014 found that 47.7% of participants were LP, and 29.3% were AHD (8). Another study found that the LP prevalence in Europe between 2010 and 2016 varied considerably by region, from 64.2% in central Europe to 47.1% in East Europe and 48.4% in the West (17). In 2012, Tang et al. observed that 63.8% of LP-categorized HIV diagnoses were from four areas in China (Henan, Guangxi, Guangdong, and Yunnan) (18). The rate of LP remained high and stable for many years in many cohort studies. One study, conducted in Canada from 1999 to 2013, found that the prevalence of LP varied slightly from 50.9 to 57.4% (19). Results from a multicenter cohort study during 2004–2018 revealed that the prevalence of LP decreased from 51.8% (2004–2008) to 40.9% (2009–2012), then remained stable at 42.0% during 2013 to 2018 in Spain (20). However, information on the long-term change in LP and AHD trends, a valuable indicator for HIV testing promotion programs, is limited in China. A study in southwestern China reported a 5-year trend on LP and AHD and saw that the percentage of LP and AHD remained stable at 70.2 and 45.1%, respectively (2).

Jiangsu, located in eastern China, belongs to the Yangtze-river economic zone, one of China's three the most developed areas, but this zone's data on LP and AHD over a long period is limited. Our study findings may be more representative in showcasing the LP and AHD trends since the duration span over 13 years and were conducted within the Yangtze-river economic zone. Understanding the LP and AHD situations and trends is essential in guiding future HIV prevention and care programs in Jiangsu province. So, the purpose of this study was to firstly assess the rate trends of LP and AHD in Jiangsu from 2008 to 2020, and secondly identify the potential driving forces of LP and AHD to provide evidence for decreasing the rate of LP and AHD in the province.

## Materials and methods

### Ethical statement

This was a serial cross-sectional study using annual data extracted from the web-based Comprehensive Response Information Management System (CRIMS). CRIMS is a real-time case reporting system for all notifiable infectious diseases in China. For HIV/AIDS, the newly diagnosed people will get into the healthcare system, and be recorded sequential information using a standard questionnaire for every follow-up visit. The eligible data were de-identified before the data analysis. The study's process and contents were reviewed and approved by the National Center for AIDS/STD Control and Prevention, Chinese Center for Disease Control and Prevention (No. of IRB Application: X140121318).

### Study population

We included data on newly diagnosed PLWH registered in the CRIMS in Jiangsu Provincial Center for Disease Control and Prevention between January 2008 and December 2020. The inclusion criteria were as follows: (1) HIV positive, (2) aged at least 15 years, (3) had a CD4^+^ *T*-cell count test within 6 months after HIV diagnosis, (4) ART-naïve when the first CD4^+^ *T*-cell count was detected.

### Study design

Information was collected using the standard surveillance forms on the following variables: socio-economic data, including age at HIV diagnosis, gender, ethnicity, level of education, marital status, migration status, and history of sexually transmitted infections (STIs). We determined migratory status based on patients' official household registrations. Patients who did not have official household registration in Jiangsu when first diagnosed with HIV were deemed immigrants (21). We also recorded the information on ethnicity from the identification card, a standard definition in the census.

The information of route of HIV infection was judged by professional clinicians when patients were diagnosed HIV infection firstly based on self-report risk behaviors. There are four types of route transmission, named as homosexual, heterosexual, inject drug user (IDU), others did not disclose their potential transmission routes. We classified routes of HIV infection as homosexual, heterosexual, IDU and others in this study.

Clinical data also recorded by CRIMS, included: the year of HIV diagnosis, CD4^+^ *T*-cell count after HIV diagnosis and place of HIV testing.

LP in this study referred to patients having the first-ever CD4 testing (<350cells/mm^3^) or presented a AIDS-defining illness regardless of the CD4^+^ *T*-cell count during HIV diagnosis. If patients had the first-ever CD4 testing (≥350 cells/mm^3^) during HIV diagnosis, we defined these patients as non-LP. AHD was defined as patients had first-ever CD4^+^ *T*-cell count (<200 cells/mm^3^) or presented a AIDS-defining illness regardless of the CD4^+^ *T*-cell count during HIV diagnosis (22). On the contrary, if patients had the first-ever CD4 testing (≥200 cells/mm^3^) during HIV diagnosis, we defined these patients as non-AHD.

### Statistical analysis

Join-point regression was estimated for general and transmission group to identify changes in the proportion of LP and AHD trends and the median CD4 levels. This analysis was done using the Join-point regression Program, Version 4.9.1.0 (Statistical Research and Applications Branch, National Cancer Institute). In brief, by using LP and AHD proportions and median of CD4 as inputs, this method identifies the years when a trend change is produced. It calculates the annual percentage change (APC) between trend-change points. It also estimates the average annual percentage (APPC) in the whole period. We obtained the number of join points via Monte Carlo resampling (23). Based on the disparities of variables, a maximum number of two points was allowed, ensuring the results were credible. We choose the exact join points owing to the smallest Bayesian Information Criterion (24). All results were presented in the Supplementary material.

We assessed factors associated with LP and AHD in a univariate logistic regression, firstly. Variables with *P*-values <0.2 were entered into a multivariable logistic regression model. Results were presented based on odds ratios (OR) and their 95% confidence interval (CI). All analyses were performed using IBM SPSS STATISTICS (version 19.0, SPSS Inc., Chicago, IL, USA).

## Results

### Characteristics of LP and AHD

**Figure 1:**
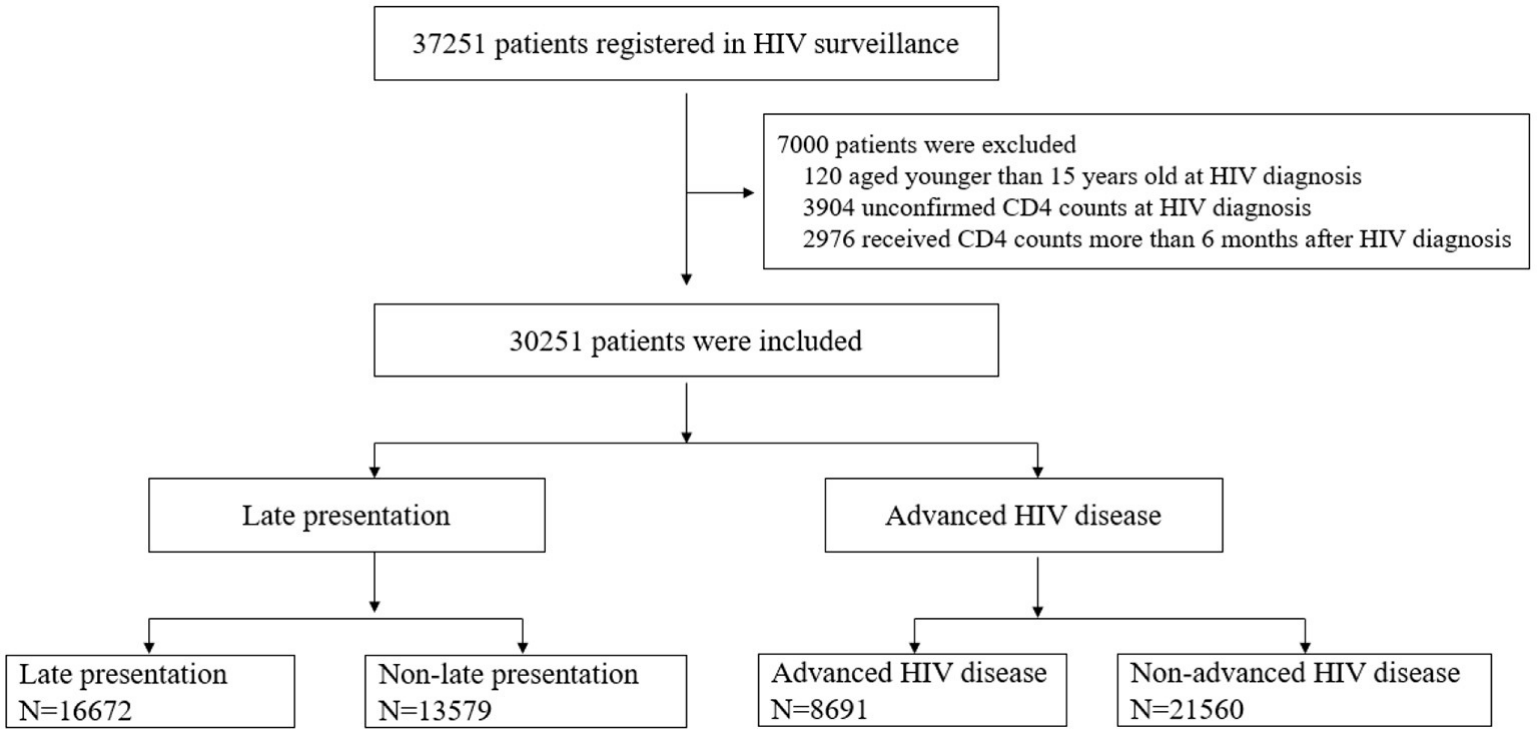
Chart of the inclusion and exclusion criteria in this study.

In total, 37,251 patients were identified during the study period, of which 30,251(81.2%) met the inclusion criteria. 7,000(18.8%) patients were excluded from the study. Among the excluded patients, 120(0.3%) were below 15 years old at first HIV diagnosis, and 3,904(10.5%) PLWH did not receive CD4^+^ *T*-cell count tests after the first HIV diagnosis. 2,976(8.0%) PLWH did not receive CD4^+^ *T*-cell count within 6 months after HIV diagnosis. The median age of the included cases was 38 years old (IQR: 26–49). Further details are in Figure 1.

Overall, 16,672 (55.1%) patients were LP, and 8,691 (28.7%) were AHD based on the 2008 to 2020 retrieved study data. The overall rate trends of LP and AHD were presented in Supplementary Table 1.

Most LP patients were <35 years old (37.6%), of Han ethnicity (98.6%), residents of Jiangsu (73.8%), with primary education or below (51.1%), and married (53.0%). Additionally, 52.8% of LP patients became HIV infected via homosexual transmission, 46.8% were diagnosed in hospitals, and 26.4% received their first-ever diagnosis at voluntary counseling and testing (VCT) center. However, LP was high among hospital diagnosed patients (67.0%) (Table 1).

**Table 1:** Demographic and sociological features of the HIV/AIDS patients in China from 2008 to 2020.

**Characteristics at HIV diagnosis**	**Total *N* (%)**	**Late presentation *N* (%)**	**Non-late presentation *N* (%)**	**Advanced HIV disease *N* (%)**	**Non-advanced HIV disease *N* (%)**
**Age (year)**					
<35	14,523 (48.0)	6,274 (37.6)	8,249 (60.7)	2,784 (32.0)	11,739 (54.4)
35–49	8,435 (27.9)	5,183 (31.1)	3,252 (23.9)	2,975 (34.2)	5,460 (25.3)
≥50	7,293 (24.1)	5,215 (31.3)	2,078 (15.3)	2,932 (33.7)	4,361 (20.2)
**Gender**					
Male	26,575 (87.8)	14,515 (87.1)	12,060 (88.8)	7,654 (88.1)	18,921 (87.8)
Female	3,676 (12.2)	2,157 (12.9)	1,519 (11.2)	1,037 (11.9)	2,639 (12.2)
**Ethnicity**					
Han	29,698 (98.2)	16,442 (98.6)	13,256 (97.6)	8,596 (98.9)	21,102 (97.9)
Minority	553 (1.8)	230 (1.4)	323 (2.4)	95 (1.1)	458 (2.1)
**Immigrant**					
Yes	9,530 (31.5)	4,365 (26.2)	5,165 (38.0)	1,892 (21.8)	7,638 (35.4)
No	20,721 (68.5)	12,037 (73.8)	8,414 (62.0)	6,799 (78.2)	13,922 (64.6)
**Education**					
Primary/No education	13,902 (46.0)	8,524 (51.1)	5,378 (39.6)	4,656 (53.6)	9,246 (42.9)
Secondary education	7,350 (24.3)	3,863 (23.2)	3,487 (25.7)	2,050 (23.6)	5,300 (24.6)
University	8,999 (29.7)	4,285 (25.7)	4,714 (34.7)	1,985 (22.8)	7,014 (32.5)
**Marital status**					
Single	11,764 (38.9)	5,130 (30.8)	6,634 (48.9)	2,279 (26.2)	9,485 (44.0)
Married	13,952 (46.1)	8,833 (53.0)	5,119 (37.7)	4,911 (56.5)	9,041 (41.9)
Divorced/widowed	4,535 (15.0)	2,709 (16.2)	1,826 (13.4)	1,501 (17.3)	3,034 (14.1)
**History of STI**					
Yes	4,724 (15.6)	2,613 (15.7)	2,111 (15.5)	7,317 (84.2)	18,210 (84.5)
No	25,527 (84.4)	14,059 (84.3)	11,468 (84.5)	1,374 (15.8)	3,350 (15.5)
**Route of HIV infection**					
Homosexual	17,212 (56.9)	8,805 (52.8)	8,407 (61.9)	4,224 (48.6)	12,988 (60.2)
Heterosexual	12,406 (41.0)	7,553 (45.3)	4,853 (35.7)	4,293 (49.4)	8,113 (37.6)
IDU	332 (1.1)	116 (0.7)	216 (1.6)	51 (0.6)	281 (1.3)
Others	301 (1.0)	198 (1.2)	103 (0.8)	123 (1.4)	178 (0.8)
**Reason for HIV testing**					
VCT	9,188 (30.4)	4,407 (26.4)	4,781 (35.2)	1,848 (21.3)	7,340 (34.0)
STI clinics	2,931 (9.7)	1,475 (8.8)	1,456 (10.7)	680 (7.8)	2,251 (10.4)
Premarital/pregnancy screening	597 (2.0)	253 (1.5)	344 (2.5)	82 (0.9)	515 (2.4)
Penitentiary	520 (1.7)	191 (1.1)	329 (2.4)	69 (0.8)	451 (2.1)
Medical examination	242 (0.8)	92 ()0.6	150 (1.1)	32 (0.4)	210 (1.0)
Hospital	11,653 (38.5)	7,803 (46.8)	3,850 (28.4)	4,910 (56.5)	6,743 (31.3)
Blood testing of blood donation/receivers	1,253 (4.1)	564 (3.4)	689 (5.1)	241 (2.8)	1,012 (4.7)
Event-specific survey	2,054 (6.8)	851 (5.1)	1,203 (8.9)	318 (3.7)	1,736 (8.1)
HIV positive couple/partners	1,071 (3.5)	639 (3.8)	432 (3.2)	267 (3.1)	804 (3.7)
Others	742 (2.5)	397 (2.4)	345 (2.5)	244 (2.8)	498 (2.3)
**Year of diagnosis**					
2008	254 (0.8)	99 (0.6)	155 (1.1)	25 (0.3)	229 (1.1)
2009	443 (1.5)	223 (1.3)	220 (1.6)	127 (1.5)	316 (1.5)
2010	788 (2.6)	370 (2.2)	418 (3.1)	223 (2.6)	565 (2.6)
2011	1,153 (3.8)	587 (3.5)	566 (4.2)	365 (4.2)	788 (3.7)
2012	1,734 (5.7)	808 (4.8)	926 (6.8)	522 (6.0)	1,212 (5.6)
2013	2,252 (7.4)	1,127 (6.8)	1,125 (8.3)	719 (8.3)	1,533 (7.1)
2014	2,836 (9.4)	1,436 (8.6)	1,400 (10.3)	829 (9.5)	2,007 (9.3)
2015	3,172 (10.5)	1,706 (10.2)	1,466 (10.8)	1,028 (11.8)	2,144 (9.9)
2016	3,243 (10.7)	1,803 (10.8)	1,440 (10.6)	1,046 (12.0)	2,197 (10.2)
2017	3,421 (11.3)	1,984 (11.9)	1,437 (10.6)	941 (10.8)	2,480 (11.5)
2018	3,643 (12.0)	2,176 (13.1)	1,467 (10.8)	1,015 (11.7)	2,628 (12.2)
2019	3,810 (12.6)	2,272 (13.6)	1,538 (11.3)	1,032 (11.9)	2,778 (12.9)
2020	3,502 (11.6)	2,081 (12.5)	1,421 (10.5)	819 (9.4)	2,683 (12.4)

STI, sexually transmitted infection; IDU, inject drug users; VCT, voluntary counseling and testing.

Among AHD patients, 33.7% were over 50 years, 98.9% were of Han ethnicity, 78.2% were residents of Jiangsu province, 53.6% had primary education or below, and 56.5% were married. Most AHD patients became HIV infected through heterosexual transmissions (49.4%), and AHD was more prominent among patients diagnosed in hospitals (56.5%) (Table 1).

### Trends of LP and AHD by transmission routes

**Figure 2:**
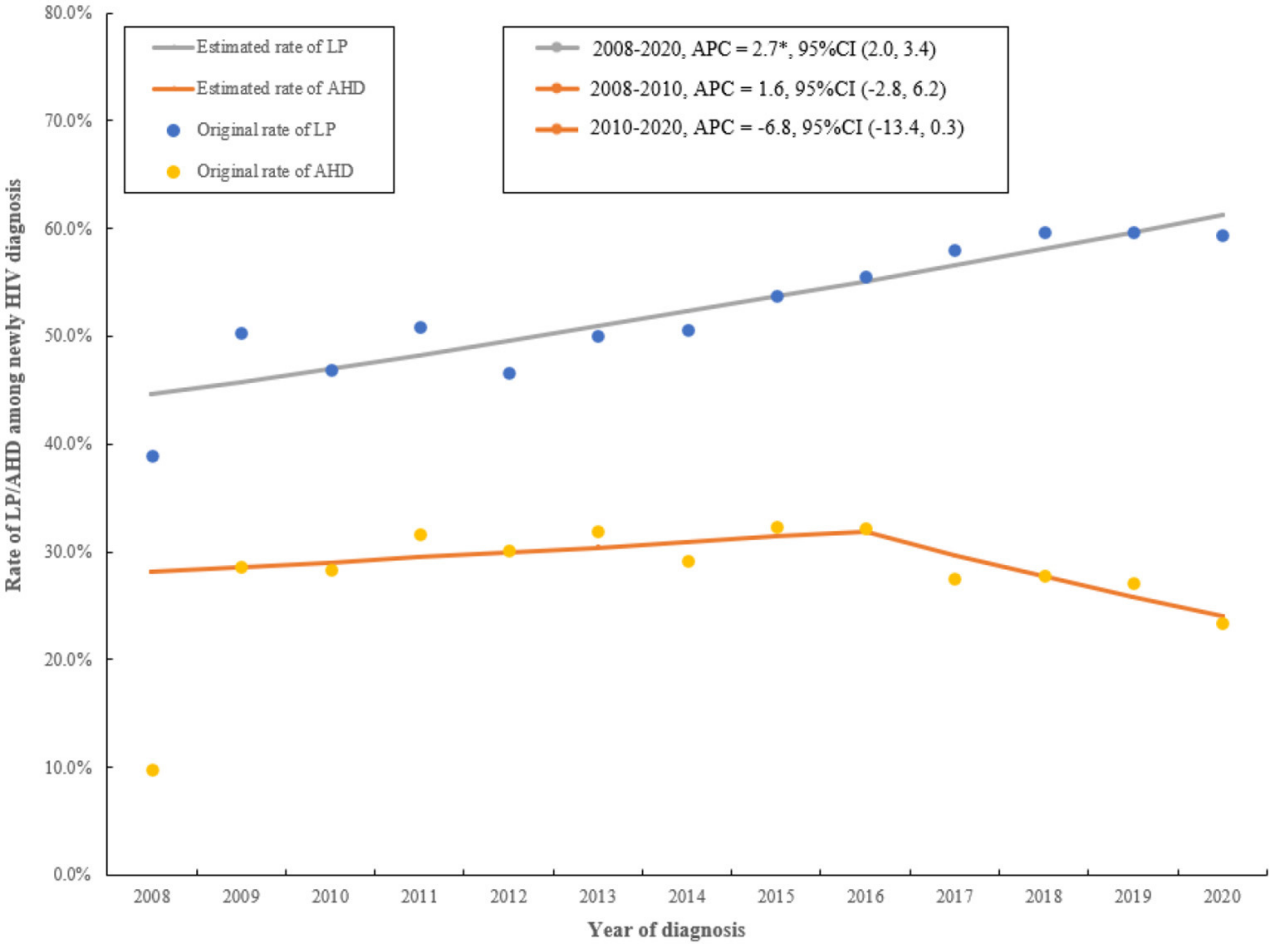
The trends of original and estimated rate for LP and AHD from 2008 to 2020, in Jiangsu, China. LP, late presentation; AHD, advanced HIV disease; APC, annual percentage change; CI, confidence interval; *P < 0.05.

The rate of LP increased from 39.0 to 59.4% between 2008 to 2020 (APC = 2.7, 95%CI (2.0, 3.4), *P* < 0.001). The rate of AHD increased from 9.8 to 32.3% from 2008 to 2016 (APC = 1.6, 95%CI (−2.8, 6.2), *P* = 0.433), then decreased from 32.3 to 23.4% during 2016 to 2020 (APC = −6.8, 95%CI (−13.4, 0.3), *P* = 0.057) (Figure 2; Supplementary Tables 1, 5). The APPC in the whole period was 2.7 (95%CI: 2.0, 3.4) and −1.3 (95%CI: −4.5, 2.0) for LP and AHD, respectively (Supplementary Table 5).

**Figure 3:**
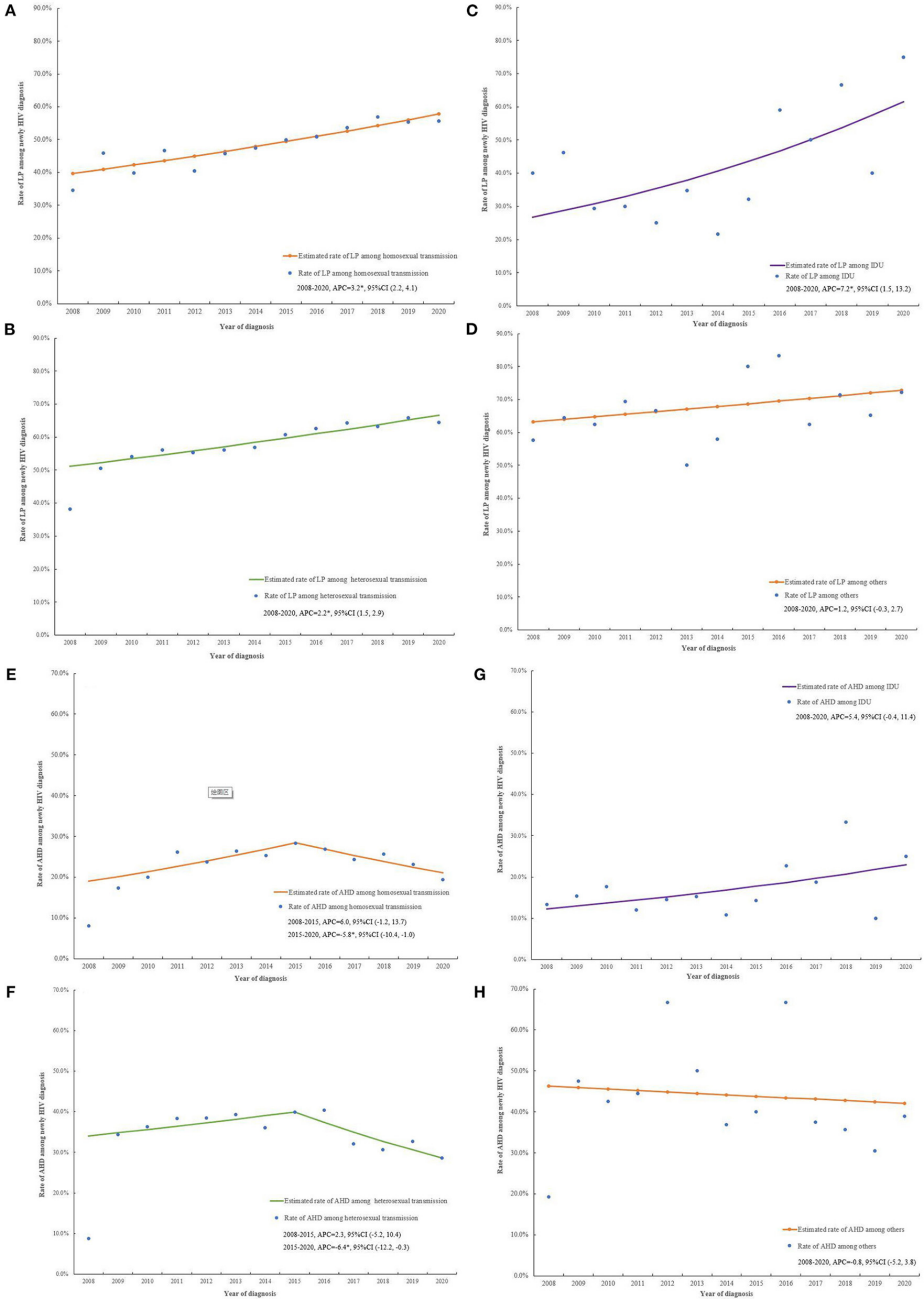
The trends of original and estimated rate for LP and AHD by transmission routes. The rate trends of LP among homosexual transmission **(A)**, heterosexual transmission **(B)**, IDU **(C)**, and others **(D)** from 2008 to 2020 in Jiangsu. The rate trends of AHD among homosexual transmission **(E)**, heterosexual transmission **(F)**, IDU **(G)**, and others **(H)** from 2008 to 2020, in Jiangsu, China. LP, late presentation; AHD, advanced HIV disease; APC, annual percentage change; CI, confidence interval; *P < 0.05.

Figure 3 and Supplementary Tables 2, 3 demonstrates the trends of LP and AHD between 2008 and 2020 by transmission groups, respectively. The rate of LP rose from 34.5 to 55.6% (APC = 3.2, 95%CI (2.2, 4.1), *P* < 0.001) (Figure 3A) for homosexual transmission, 38.1 to 64.4% (APC = 2.2, 95%CI (1.5, 2.9), *P* < 0.001) (Figure 3B) for heterosexual transmission, and 40.0 to 75.0% (APC = 7.2, 95%CI (1.5, 13.2), *P* = 0.017) (Figure 3C) for IDU between 2008 and 2020, respectively. The proportion of AHD dropped from 28.3 to 19.4% (APC = −5.8, 95%CI (−10.4, −1.0), *P* = 0.025) (Figure 3E) for homosexual transmission group, and 39.9 to 28.6% (APC = −6.4, 95%CI (−12.2, −0.3), *P* = 0.043) (Figure 3F) for heterosexual transmission during 2015 to 2020, respectively.

The APC and APPC of LP and AHD between 2008 and 2020 by transmission groups were in Supplementary Table 6.

### Trends of median CD4 level by transmission routes

**Figure 4:**
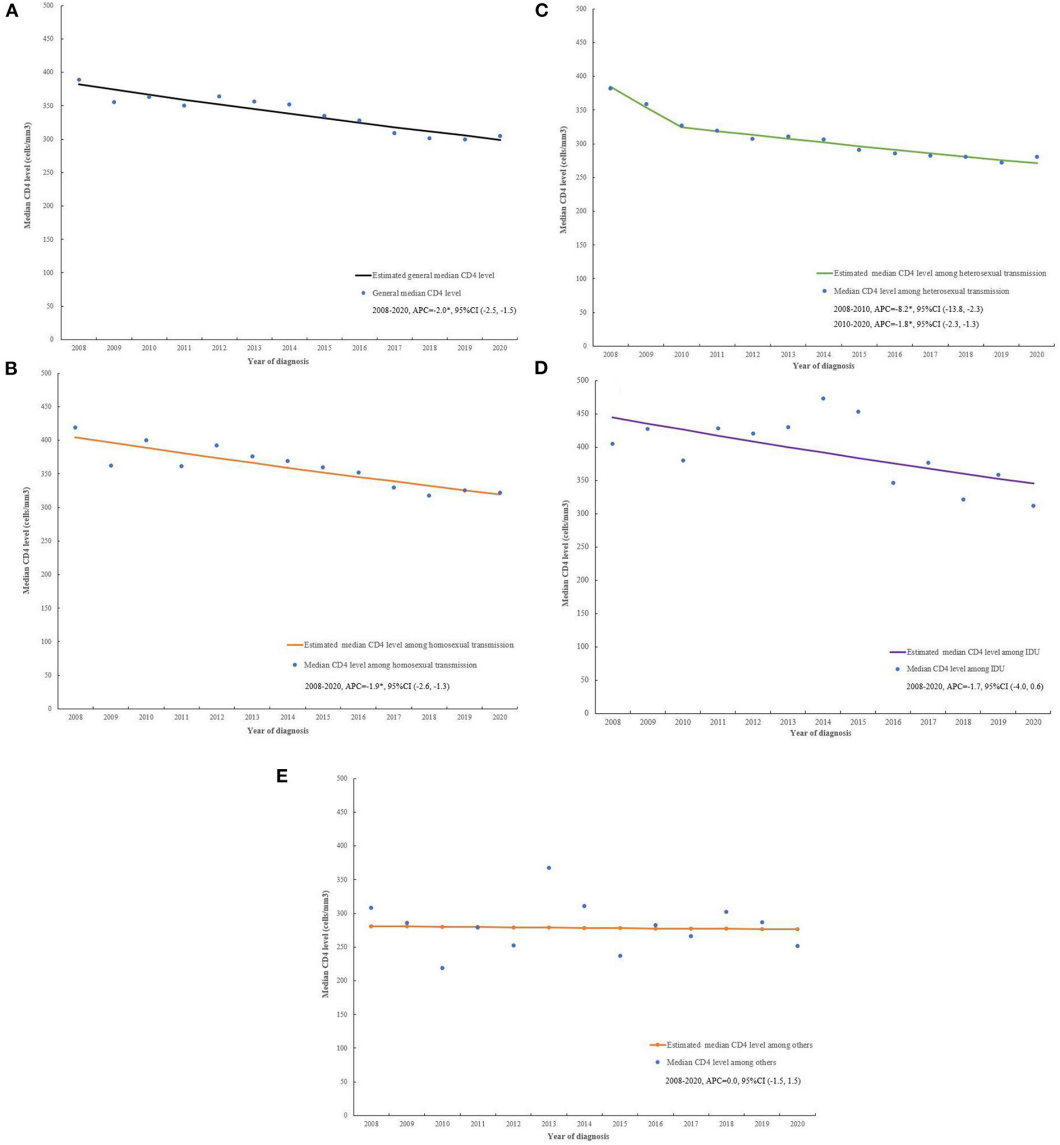
The trends of median CD4 level for general **(A)**, homosexual transmission **(B)**, heterosexual transmission **(C)**, IDU **(D)**, and others **(E)** from 2008 to 2020, in Jiangsu, China. APC, annual percentage change; CI, confidence interval; *P < 0.05.

The general median CD4 level dropped from 389 to 305 cells/mm^3^ (APC = −2.0, 95%CI (−2.5, −1.5), *P* < 0.001) between 2008 and 2020 (Figure 4A, Supplementary Table 4). The median CD4 level dropped from 419 to 322 cells/mm^3^ (APC = −1.9, 95%CI (−2.6, −1.3), *P* < 0.001) (Figure 4B) for homosexual transmission, 405 to 312 cells/mm^3^ (APC = −1.7, 95%CI (−4.0, 0.6), *P* = 0.132) for IDU (Figure 4D), 308 to 252 cells/mm^3^ (APC = 0.0, 95%CI (−1.5, 1.5), *P* = 0.998) for others (Figure 4E) between 2008 and 2020, respectively. For heterosexual transmission, median CD4 level decreased slightly from 327 to 281 cells/mm^3^ (APC = −1.8, 95%CI (−2.3, −1.3), *P* < 0.001) during 2010 and 2020 (Figure 4C).

The APC and APPC of median CD4 levels by general and transmission groups between 2008 and 2020 were presented in Supplementary Tables 7, 8.

### Factors associated with LP

In the univariate model, risk factors associated with LP were being older than 35 years, female, of Han ethnicity, married or divorced/widowed, getting HIV from heterosexual transmission, diagnosed at STIs clinics or hospitals, having partners living with HIV, diagnosed after 2008 (Table 2).

**Table 2:** Risk factors associated with late HIV presentation in Jiangsu province, China, 2008–2020.

**Characteristics of HIV diagnosis**	**Univariate model (OR: 95%CI)**	***P*-value**	**Multivariable model (aOR: 95%CI)**	***P*-value**
**Age (year)**				
<35	Reference		Reference	
35–49	2.10 (1.98–2.21)	<0.001	1.72 (1.60–1.85)	<0.001
≥50	3.30 (3.11–3.51)	<0.001	2.21 (2.04–2.41)	<0.001
**Gender**				
Male	Reference		Reference	
Female	1.18 (1.10–1.27)	<0.001	0.88 (0.80–0.95)	0.002
**Ethnicity**				
Han	1.74 (1.47–2.07)	<0.001	1.12 (0.93–1.35)	0.222
Minority	Reference		Reference	
**Immigrant**				
Yes	0.58 (0.55–0.61)	<0.001	0.84 (0.80–0.89)	<0.001
No	Reference		Reference	
**Education**				
Primary/no education	Reference		Reference	
Secondary education	0.70 (0.66–0.74)	<0.001	0.94 (0.88–1.00)	0.037
University	0.57 (0.54–0.61)	<0.001	1.00 (0.93–1.06)	0.875
**Marital status**				
Single	Reference		Reference	
Married	2.23 (2.12–2.35)	<0.001	1.25 (1.17–1.35)	<0.001
Divorced/widowed	1.92 (1.79–2.06)	<0.001	1.10 (1.01–1.19)	0.037
**History of STI**				
Yes	1.01 (0.95–1.08)	0.762		
No	Reference			
**Route of HIV infection**				
Homosexual	Reference		Reference	
Heterosexual	1.49 (1.42–1.56)	<0.001	0.98 (0.92–1.04)	0.459
IDU	0.51 (0.41–0.64)	<0.001	0.66 (0.52–0.85)	0.001
Others	1.84 (1.44–2.33)	<0.001	1.48 (1.14–1.91)	0.003
**Reason for HIV testing**				
VCT	Reference		Reference	
STI clinics	1.10 (1.01–1.19)	0.026	1.10 (1.01–1.20)	0.030
Premarital/pregnancy screening	0.80 (0.68–0.94)	0.008	0.96 (0.81–1.15)	0.673
Penitentiary	0.63 (0.53–0.76)	<0.001	0.79 (0.64–0.96)	0.020
Medical examination	0.67 (0.51–0.87)	0.002	0.60 (0.46–0.79)	<0.001
Hospital	2.20 (2.08–2.33)	<0.001	1.69 (1.59–1.79)	<0.001
Blood testing of blood donation/receivers	0.89 (0.79–1.00)	0.050	0.89 (0.79–1.00)	0.059
Event-specific survey	0.77 (0.70–0.85)	<0.001	0.86 (0.78–0.95)	0.003
HIV positive couple/partners	1.61 (1.41–1.83)	<0.001	1.10 (0.96–1.27)	0.183
Others	1.25 (1.08–1.45)	<0.001	1.19 (1.02–1.39)	0.027
**Year of diagnosis**				
2008	Reference		Reference	
2009	1.59 (1.16–2.17)	0.004	1.52 (1.10–2.11)	0.011
2010	1.39 (1.04–1.85)	0.027	1.34 (0.99–1.81)	0.057
2011	1.62 (1.23–2.14)	0.001	1.58 (1.18–2.10)	0.002
2012	1.37 (1.04–1.79)	0.023	1.31 (0.99–1.74)	0.058
2013	1.57 (1.20–2.05)	0.001	1.45 (1.10–1.92)	0.008
2014	1.61 (1.24–2.09)	<0.001	1.53 (1.16–2.01)	0.002
2015	1.82 (1.10–2.37)	<0.001	1.75 (1.33–2.30)	<0.001
2016	1.96 (1.51–2.55)	<0.001	1.84 (1.40–2.41)	<0.001
2017	2.16 (1.67–2.81)	<0.001	1.95 (1.48–2.56)	<0.001
2018	2.32 (1.79–3.01)	<0.001	2.03 (1.54–2.66)	<0.001
2019	2.31 (1.78–3.00)	<0.001	2.01 (1.53–2.64)	<0.001
2020	2.29 (1.77–2.98)	<0.001	1.94 (1.48–2.55)	<0.001

STI, Sexually transmitted infection; IDU, Inject Drug Users; VCT, Voluntary Counseling and Testing; OR, Odds ratios; CI, Confidence interval.

In the multivariable model, risk factors associated with LP were: being older than 35 (35–49: aOR: 1.72,95%CI: 1.60–1.85; *P* < 0.001; ≥50 years old: aOR: 2.21, 95%CI: 2.04–2.41; *P* < 0.001) and being married (aOR: 1.25, 95%CI: 1.17–1.35; *P* < 0.001). Additionally, patients diagnosed at STIs clinics (aOR: 1.10, 95%CI: 1.01–1.20; *P* = 0.030), and hospitals (aOR: 1.69, 95%CI: 1.59–1.79; *P* < 0.001) tended to be LP. On the contrary, immigrants (aOR: 0.84, 95%CI: 0.80–0.89; *P* < 0.001), received secondary education (aOR: 0.94, 95%CI: 0.88–1.00; *P* = 0.037), transmitted by IDU (aOR: 0.66, 95%CI: 0.52–0.85; *P* = 0.001) were less likely to LP. Meanwhile, PLWH diagnosed at penitentiary sites (aOR: 0.79, 95%CI: 0.64–0.96; *P* = 0.020), or medical examination sites (aOR: 0.60, 95%CI: 0.46–0.79; *P* < 0.001), or event-specific survey (aOR: 0.86, 95%CI: 0.78–0.95; *P* = 0.003) were less likely to LP (Table 2).

Being female were tended to be LP in univariate model, but turned to be a protective factor in multivariable model. PLWH were of Han ethnicity, received university education, getting HIV from heterosexual transmission, diagnosed by premarital or pregnancy screening were tended to be LP in univariate model, but these factors did not confirm as risk factors in multivariable model (Table 2).

### Factors associated with AHD

In the univariate model, patients older than 35, of Han ethnicity, and married or divorced/widowed, infected through heterosexual transmission, obtained HIV tests at STIs clinics or hospital, having partners living with HIV, and diagnosed after 2008 tended to be AHD (Table 3).

**Table 3:** Risk factors associated with advanced HIV disease presentation in Jiangsu province, China, 2008–2020.

**Characteristics at HIV diagnosis**	**Univariate model (OR: 95%CI)**	***P*-value**	**Multivariable model (aOR: 95%CI)**	***P*-value**
**Age (year)**				
<35	Reference		Reference	
35–49	2.30 (2.16–2.44)	<0.001	1.75 (1.62–1.90)	<0.001
≥50	2.84 (2.66–3.02)	<0.001	1.80 (1.65–1.97)	<0.001
**Gender**				
Male	Reference		Reference	
Female	0.97 (0.90–1.05)	0.458	0.71 (0.65–0.78)	<0.001
**Ethnicity**				
Han	1.96 (1.57–2.45)	<0.001	1.16 (0.92–1.47)	0.208
Minority	Reference		Reference	
**Immigrant**				
Yes	0.51 (0.48–0.54)	<0.001	0.76 (0.71–0.81)	<0.001
No	Reference		Reference	
**Education**				
Primary/No education	Reference		Reference	
Secondary education	0.77 (0.72–0.82)	<0.001	1.00 (0.94–1.07)	0.974
University	0.56 (0.53–0.60)	<0.001	1.01 (0.94–1.09)	0.728
**Marital status**				
Single	Reference		Reference	
Married	2.26 (2.14–2.39)	<0.001	1.23 (1.14–1.34)	<0.001
Divorced/widowed	2.06 (1.91–2.22)	<0.001	1.13 (1.03–1.24)	0.014
**History of STI**				
Yes	1.02 (0.95–1.09)	0.556		
No	Reference			
**Route of HIV infection**				
Homosexual	Reference		Reference	
Heterosexual	1.63 (1.55–1.71)	<0.001	1.13 (1.06–1.21)	<0.001
IDU	0.56 (0.41–0.75)	<0.001	0.66 (0.48–0.92)	0.014
Others	2.13 (1.68–2.68)	<0.001	1.65 (1.28–2.13)	<0.001
**Reason for HIV testing**				
VCT	Reference		Reference	
STI clinics	1.20 (1.09–1.33)	<0.001	1.23 (1.11–1.36)	<0.001
Premarital/pregnancy screening	0.63 (0.50–0.80)	<0.001	0.71 (0.55–0.91)	0.007
Penitentiary	0.61 (0.47–0.79)	<0.001	0.68 (0.52–0.89)	0.006
Medical examination	0.61 (0.42–0.88)	0.009	0.45 (0.31–0.66)	<0.001
Hospital	2.90 (2.72–3.08)	<0.001	2.27 (2.12–2.43)	<0.001
Blood testing of blood donation/receivers	0.95 (0.82–1.10)	0.466	0.84 (0.72–0.98)	0.025
Event-specific survey	0.73 (0.64–0.83)	<0.001	0.76 (0.66–0.86)	<0.001
HIV positive couple/partners	1.32 (1.14–1.53)	<0.001	0.98 (0.84–1.15)	0.830
Others	1.95 (1.66–2.29)	<0.001	1.69 (1.43–2.00)	<0.001
**Year of diagnosis**				
2008	Reference		Reference	
2009	3.68 (2.32–5.84)	<0.001	3.51 (2.18–5.65)	<0.001
2010	3.62 (2.33–5.62)	<0.001	3.57 (2.26–5.62)	<0.001
2011	4.24 (2.76–6.53)	<0.001	4.06 (2.60–6.33)	<0.001
2012	3.95 (2.58–6.04)	<0.001	3.78 (2.44–5.87)	<0.001
2013	4.30 (2.82–6.55)	<0.001	3.91 (2.53–6.04)	<0.001
2014	3.78 (2.48–5.76)	<0.001	3.51 (2.28–5.42)	<0.001
2015	4.39 (2.89–6.68)	<0.001	4.19 (2.72–6.45)	<0.001
2016	4.36 (2.87–6.63)	<0.001	4.00 (2.59–6.16)	<0.001
2017	3.48 (2.29–5.29)	<0.001	2.99 (1.94–4.61)	<0.001
2018	3.54 (2.33–5.38)	<0.001	2.92 (1.89–4.49)	<0.001
2019	3.40 (2.24–5.17)	<0.001	2.81 (1.82–4.33)	<0.001
2020	2.80 (1.84–4.26)	<0.001	2.21 (1.44–3.43)	<0.001

STI, sexually transmitted infection; IDU, inject drug users; VCT, voluntary counseling and testing; OR, odds ratios; CI, confidence interval.

In the multivariable model, factors associated with AHD were: older than 35(35–49: aOR: 1.75,95%CI: 1.62–1.90; *P* < 0.001; ≥50 years old: aOR: 1.80, 95%CI: 1.65–1.97; *P* < 0.001), and married (aOR: 1.23, 95%CI: 1.14–1.34; *P* < 0.001) or divorced/widowed (aOR: 1.13, 95%CI: 1.03–1.24; *P* = 0.014). Patients infected via heterosexual transmission (aOR: 1.13, 95%CI: 1.06–1.21; *P* < 0.001) were more likely to be AHD. Patients diagnosed in STIs clinics (aOR: 1.23, 95%CI: 1.11–1.36; *P* < 0.001), hospital (aOR: 2.27, 95%CI: 2.12–2.43; *P* < 0.001) tended to be AHD. However, PLWH were immigrants (aOR: 0.76, 95%CI: 0.71–0.81; *P* < 0.001), transmitted by IDU (aOR: 0.66, 95%CI: 0.48–0.92; *P* = 0.014) less likely to have AHD. Meanwhile, PLWH received HIV testing from premarital or pregnancy screening sites (aOR: 0.71, 95%CI: 0.55–0.91; *P* = 0.007), penitentiary sites (aOR: 0.68, 95%CI: 0.52–0.89; *P* = 0.006), medical examination (aOR: 0.45, 95%CI: 0.31–0.66; *P* < 0.001), blood testing of blood donation (aOR: 0.84, 95%CI: 0.72–0.98; *P* = 0.025), or event-specific survey (aOR: 0.76, 95%CI: 0.66–0.86; *P* < 0.001) were less likely to have AHD (Table 3).

PLWH were of Han ethnicity, received secondary or university education, and having seroconvert partners were tended to have AHD in univariate model, but these factors did not confirm as risk factors in multivariable model (Table 3).

## Discussion

In this serial cross-sectional study, we enrolled 81.2% (25, 26) of all newly diagnosed PLWH registered in the CRIMS in Jiangsu province. We observed that the percentage of LP and AHD was high and posed an alarming problem to HIV prevention and control interventions in Jiangsu. More than 50% of patients were LP, and nearly one third were AHD patients. The AHD trend has visibly decreased since 2016. However, LP trend increased steadily from 2008 to 2020 during the study period, indicating that LP was a consistent problem in Jiangsu, China. LP may be due to HIV testing policies and the complexities of transmission dynamics, including complicated high-risk subgroups, interlaced and multiple transmission routes in Jiangsu.

The coverage disparity in HIV testing between high-risk and general population might facilitate the increasing trend of LP. The Chinese government initiated a policy, “the Five expand and six strengthen,” to cope with the increasing challenges since 2010 (25). With the “Five Expands, Six Strengthens” policy, HIV testing increased from 4.87 million to 10.92 million in the study period in Jiangsu. However, most HIV testing was facility-based as testing pre-surgery and in-patient, which is a mandatory test in hospitals, accounted for 56% of testing recorded. A high rate of LP implies a gap in accessibility to testing services or that people lack the willingness or sensitivity for HIV testing, especially among the general population (27–30). Previous studies reported an HIV testing coverage of 77.3% for men who have sex with men (MSM), 76.7% for female sex workers, and 53.8% for drug users, among high-risk populations (26, 31). At the same time, the prevalence of any HIV testing service uptake in a lifetime was only 25.2% among the general population (32). In addition, proportion of HIV diagnosed cases over 50 years old has increased steadily, which may facilitate the increasing trend of LP. The proportion of sexual transmission has been the dominant route of HIV transmission since 2007 (33), and the more and more evidence showed increased HIV prevalence among older adults compared with the general population. The knowledge of HIV/AIDS prophylaxis in this population group was lower than other population groups in China (34, 35). Meanwhile, the 86.2% rate of unprotected sexual activities among older adults, especially during commercial sex, was much higher than in the younger population (36–38). Also, our study findings indicated that older age was a risk factor for LP. Hence, the government must allocate more resources to increase HIV awareness in the general population and encourage provider-initiated HIV testing and counseling (PITC) to facilitate early HIV diagnosis.

AHD trend varied slightly over years since 2009. Then, AHD trend declined visibly during 2016 and 2020. The declining AHD trend is encouraging and may be attributed to the successful progress of multiple HIV interventions, such as HIV testing expansion, treat all policy, continual improvement on linkage to care and awareness of AIDS prevention work. Evidence shows that the rate of new HIV infections is declining nationwide in China (39, 40). However, the number of newly diagnosed HIV infections still increases annually, which means that many undiagnosed HIV cases exist (41). So, the Chinese government encouraged regular HIV testing every 3–6 months among high-risk population. With this policy, HIV testing coverage in the past 12 months increased from 43.7 to 64.4% from 2009 to 2022, which gave them more opportunities for early diagnosis (31, 42). For general population, the government had initiated PITC for older who attend hospital for medical examination and patients who attend STIs clinics. Based on the subgroup analysis in this study, the trend of AHD decreased visibly in the subpopulation aged older than 50 years, signified the amazing progress on the HIV testing expansion. Followed the contrasting trends of LP and AHD, the policy to expand HIV testing may have provided more chances to enroll the concealed sub-population who were less willing to get tested but aware of possible HIV infection based on the progression of CD4^+^ *T*-cell count.

Additionally, China's ART policy has undergone a series of adjustments over the study period. From 2008 to 2009, the indicator for free ART initiation was CD4^+^ *T*-cell count of <200 cells/mm^3^ or advanced AIDS status. That changed to CD4^+^ *T*-cell count <350 cells/mm^3^ from 2010 to 2013, and <500 cells/mm^3^ between 2014 to 2015. Currently, treat all without considering the CD4^+^ *T*-cell count since 2016 (43). As it overlapped with the 'immediate treatment' policy, AHD trends decreased visibly from 32.3 to 23.4% from 2016 to 2020. The variations in AHD trends following the ART policy suggest that the treatment policy encouraged the presentation of hidden HIV infections. Meanwhile, the chain of linkage to care continued improving in China. In China, multiple steps might interrupt the linkage chain, such as confirmatory testing, obtaining HIV testing result, being evaluated ART eligibility, and receiving ART and care at designated hospital (44). One study found that 21% of MSM with a HIV positive screening test did not receive confirmatory testing in 2014 (45). With trained peers providing point-of-care testing and case management, nearly 0.2% of MSM lost to receive confirmatory testing (46). Meanwhile, center of “one-stop service,” might short duration between HIV diagnosis and linkage to care, also improve the healthcare system service (47). Compared with prior to implementing one-stop service, the coverage rate of CD4 testing rose by 69.2%, and proportion of ART initiation rose by 3 times within 30 days after HIV diagnosed (48). Furthermore, the risk of opportunistic infection increased with CD4 decline, which may have given patients more prompting to seek healthcare and diagnosis. The decreasing AHD trend may also signify the benefits of declining HIV-related disease burden, such as opportunistic infections, morbidity, and mortality.

In this study, LP and AHD trends among persons infected via homosexual transmission were always lower than the heterosexual transmission. A few reasons could explain this phenomenon. First, the coverage of HIV testing in the last 12 months among homosexuals and other high-risk groups has increased rapidly over the past few years in Jiangsu (49, 50), which give them more opportunities for early diagnosis. Our data also showed that the section of homosexuals diagnosed with HIV within the given period was younger than the group of diagnosed heterosexuals. Young homosexual patients were more likely to know about HIV prevention and HIV testing (29). Also, provisions like training by community-based organizations enable peers to provide rapid HIV testing with social support and case management also expanded HIV testing and maybe decreased the chance of LP since 2014 in Jiangsu (46). However, there were no effective strategies for HIV testing among heterosexuals in China. The success in expanding HIV testing in homosexuals can give some clues to HIV testing strategies to reduce LP rates.

The results in this study are consistent with previous literatures finding that women are less likely than men to be LP or AHD (19, 47). One possible reason was that women have a high likelihood of being tested in their lifetime; for example, they are tested during routine prenatal testing or gynecological follow-up (48). Another explanation was that women who get infected with HIV experience a slower disease development than men (49). However, men do not have enough resources or reasons for HIV testing, especially in their young age. Lack of opportunities to get HIV testing might led to LP and AHD.

Some previous studies have identified IDU as a population at high risk of LP (12, 13). However, in our data, IDU, had a lower risk of LP. In China, drugs are the prohibited activity. When IDU are apprehended by the government, HIV testing is a mandatory test in IDU. Meanwhile, the government built many methadone maintenance therapy (MMT) facilities for drug users to help them. These MMT facilities also provide basic medical care. In addition, IDU usually have poor health, which give them more reasons to visit a hospital or some other health care facilities. In these conditions, IDU have an increased chance of being tested for HIV (50).

The disparity of LP and AHD should be concerned among different marital status. In our study, current married was identified as risk factor for LP and AHD. This finding is supported by previous researches conducted in other provinces in China (51, 52). PLWH faced a highly stigma nationwide, associated with lower rate of HIV testing and lower willingness to linkage to health care (53–55). Highly stigma might delay or inhibit presentation to HIV care (56). A cross-sectional study conducted in 2014 in China found that only half of participants disclosed their HIV status to spouses (57). Without enough social support, PLWH received ART at home also increased the risk of HIV disclosure.

Our study may have some limitations. First, 18.8% of patients did not receive CD4^+^ *T*-cell count tests or receive CD4^+^ *T*-cell count tests within 6 months after their initial HIV diagnosis. There were significant differences in the basic characteristics between included and excluded patient data. These differences could have resulted in selection bias and underestimated the overall percentages of LP and AHD. Second, most patients self-reported their high-risk behaviors. Hence, some misreporting may have occurred due to fear of stigma or misunderstanding, especially for patients who have had homosexual contact or have a history of IDU (32, 58). Third, we also lack data on CD4^+^ *T*-cell count before HIV infection in our database, since we could not evaluate the dynamic of CD4 level to correct the LP proportion based on the disparity of HIV genotypes (59). Even though, China's national guidelines of Prep and Pep were promulgated in 2021. There still some people got access to PrEP or PeP by purchasing the drug on the internet or neighboring countries, especially MSM (60). That might underestimate the rate of LP and AHD. Finally, we could not ignore the impact of COVID-19 pandemic on the continuum of HIV related healthcare system, which might overestimate the rate of LP (61). However, the sample size was large, and the study period was lengthy. The risk factors associated with LP and AHD identified in this study are consistent with the findings of previous similar studies. So, our study could be representative of the whole province.

## Conclusions

This study provides provincial representative data on LP and AHD trends among PLWH in Jiangsu. The percentage of LP was relatively high and increased steadily over the years, so we should focus more on expanding early HIV diagnosis strategies. Meanwhile, AHD trends decreasing visibly in 2016 suggest that the comprehensive intervention strategies for HIV prevention are effective and should be strengthened. Especially the awareness of HIV prophylaxis and HIV testing rates among the general population should be scaled-up through provider-initiated HIV testing and counseling methods.

## Data availability statement

The raw data supporting the conclusions of this article will be made available by the authors, without undue reservation.

## Ethics statement

The studies involving human participants were reviewed and approved by China National Center for AIDS/STD Control and Prevention and the Center for Disease Control and Prevention. Written informed consent to participate in this study was provided by the participants' legal guardian/next of kin.

## Author contributions

LS and GF had the original idea. XL, HH, TQ, YuhC, XX, YunC, ZZ, YZ, and JL collected the data. LS, WT, and YuhC analyzed the data. LS wrote the main manuscript text. All authors contributed to manuscript revision and approved the submitted version.
