# Supplementary material for: Trends of late HIV presentation and advance HIV disease among newly diagnosed HIV cases in Jiangsu, China: A serial cross-sectional study from 2008 to 2020
Source: Front Public Health. 2022 Dec 8;10:1054765. doi: 10.3389/fpubh.2022.1054765 (PMC9773559; doi:10.3389/fpubh.2022.1054765)
Supplement: Supplementary file 1 [file Data_Sheet_1.docx]

**Trends of Late HIV Presentation and Advance HIV Disease among Newly Diagnosed HIV Cases in Jiangsu, China: A serial Cross-sectional study from 2008 to 2020**

Lingen Shi^1,2^, Weiming Tang^3^, Xiaoyan Liu^1^, Haiyang Hu^1^, Tao Qiu^1^, Yuheng Chen^1^, Xiaoqin Xu^1^, Yunting Chen^1^, Zhi Zhang^1^, Ying Zhou^1^, Jing Lu^1^, Gengfeng Fu^1*^

^1^ Institute for STI and HIV Control and Prevention, Jiangsu Provincial Center for Disease Control and Prevention, Jiangsu, China

^2^ Jiangsu Key Laboratory of Molecular Medicine, Medical School, Nanjing University, Jiangsu, China

^3^ University of North Carolina Project-China, Guangzhou 510095, China. Electronic address: [Weiming_tang@med.unc.edu](mailto:Weiming_tang@med.unc.edu).

Supplement table 1. Rate and estimated rate of LP and AHD in Jiangsu, China from 2008 to 2020

| Year | 2008 | 2009 | 2010 | 2011 | 2012 | 2013 | 2014 | 2015 | 2016 | 2017 | 2018 | 2019 | 2020 |
| --- | --- | --- | --- | --- | --- | --- | --- | --- | --- | --- | --- | --- | --- |
| Rate of LP (%) | 39.0 | 50.3 | 47.0 | 50.9 | 46.6 | 50.0 | 50.6 | 53.8 | 55.6 | 58.0 | 59.7 | 59.6 | 59.4 |
| Estimated rate of LP (%) | 44.6 | 45.8 | 47.0 | 48.3 | 49.6 | 50.9 | 52.3 | 53.7 | 55.1 | 56.6 | 58.1 | 59.7 | 61.3 |
| Rate of AHD (%) | 9.8 | 28.7 | 28.3 | 31.7 | 30.1 | 31.9 | 29.2 | 32.4 | 32.3 | 27.5 | 27.9 | 27.1 | 23.4 |
| Estimated rate of AHD (%) | 28.1 | 28.6 | 29.0 | 29.5 | 29.9 | 30.4 | 30.9 | 31.4 | 31.9 | 29.7 | 27.7 | 25.8 | 24.0 |

LP: Late presentation; AHD: Advanced HIV disease.

Supplement table 2. Rate and estimated rate of LP among transmission routes in Jiangsu, China from 2008 to 2020

| Year | 2008 | 2009 | 2010 | 2011 | 2012 | 2013 | 2014 | 2015 | 2016 | 2017 | 2018 | 2019 | 2020 |
| --- | --- | --- | --- | --- | --- | --- | --- | --- | --- | --- | --- | --- | --- |
| Rate of LP among homosexual transmission (%) | 34.5 | 45.8 | 39.9 | 46.7 | 40.4 | 45.7 | 47.4 | 49.9 | 50.9 | 53.7 | 56.9 | 55.3 | 55.6 |
| Estimated rate of LP among homosexual transmission (%) | 26.8 | 28.7 | 30.8 | 33.0 | 35.3 | 37.9 | 40.6 | 43.5 | 46.6 | 50.0 | 53.6 | 57.4 | 61.6 |
| Rate of LP among heterosexual transmission (%) | 38.1 | 50.5 | 54.1 | 56.0 | 55.3 | 56.1 | 56.8 | 60.8 | 62.5 | 64.3 | 63.2 | 65.8 | 64.4 |
| Estimated rate of LP among homosexual transmission (%) | 51.1 | 52.2 | 53.4 | 54.6 | 55.8 | 57.1 | 58.3 | 59.6 | 61.0 | 62.3 | 63.7 | 65.2 | 66.6 |
| Rate of LP among IDU (%) | 40.0 | 46.2 | 29.4 | 30.0 | 25.0 | 34.8 | 21.6 | 32.1 | 59.1 | 50.0 | 66.7 | 40.0 | 75.0 |
| Estimated rate of LP among IDU (%) | 26.8 | 28.7 | 30.8 | 33.0 | 35.3 | 37.9 | 40.6 | 43.5 | 46.6 | 50.0 | 53.6 | 57.4 | 61.6 |
| Rate of LP among others (%) | 57.7 | 64.4 | 62.5 | 69.4 | 66.7 | 50.0 | 57.9 | 80.0 | 83.3 | 62.5 | 71.4 | 65.2 | 72.2 |
| Estimated rate of LP among others (%) | 63.3 | 64.0 | 64.8 | 65.5 | 66.3 | 67.1 | 67.9 | 68.7 | 69.5 | 70.3 | 71.1 | 72.0 | 72.8 |

LP: Late presentation; AHD: Advanced HIV disease; IDU: Inject drug user.

Supplement table 3. Rate and estimated rate of AHD among transmission routes in Jiangsu, China from 2008 to 2020

| Year | 2008 | 2009 | 2010 | 2011 | 2012 | 2013 | 2014 | 2015 | 2016 | 2017 | 2018 | 2019 | 2020 |
| --- | --- | --- | --- | --- | --- | --- | --- | --- | --- | --- | --- | --- | --- |
| Rate of AHD among homosexual transmission (%) | 8.0 | 17.3 | 19.9 | 26.2 | 23.7 | 26.4 | 25.3 | 28.3 | 26.9 | 24.3 | 25.7 | 23.2 | 19.4 |
| Estimated rate of AHD among homosexual transmission (%) | 19.0 | 20.1 | 21.3 | 22.6 | 23.9 | 25.4 | 26.9 | 28.5 | 26.8 | 25.3 | 23.8 | 22.4 | 21.1 |
| Rate of AHD among heterosexual transmission (%) | 8.7 | 34.4 | 36.3 | 38.4 | 38.5 | 39.3 | 36.1 | 39.9 | 40.3 | 32.1 | 30.6 | 32.6 | 28.6 |
| Estimated rate of AHD among homosexual transmission (%) | 34.0 | 34.8 | 35.6 | 36.4 | 37.3 | 38.1 | 39.0 | 39.9 | 37.4 | 35.0 | 32.7 | 30.6 | 28.7 |
| Rate of AHD among IDU (%) | 13.3 | 15.4 | 17.6 | 12.0 | 14.6 | 15.2 | 10.8 | 14.3 | 22.7 | 18.8 | 33.3 | 10.0 | 25.0 |
| Estimated rate of AHD among IDU (%) | 12.3 | 13.0 | 13.7 | 14.4 | 15.2 | 16.0 | 16.8 | 17.7 | 18.7 | 19.7 | 20.7 | 21.8 | 23.0 |
| Rate of AHD among others (%) | 19.2 | 47.5 | 42.5 | 44.4 | 66.7 | 50.0 | 36.8 | 40.0 | 66.7 | 37.5 | 35.7 | 30.4 | 38.9 |
| Estimated rate of AHD among others (%) | 46.3 | 45.9 | 45.6 | 45.2 | 44.9 | 44.5 | 44.2 | 43.8 | 43.5 | 43.1 | 42.8 | 42.4 | 42.1 |

LP: Late presentation; AHD: Advanced HIV disease; IDU: Inject drug user.

Supplement table 4. The median and estimated median CD4 level among general and transmission routes in Jiangsu, China from 2008 to 2020

| Year | 2008 | 2009 | 2010 | 2011 | 2012 | 2013 | 2014 | 2015 | 2016 | 2017 | 2018 | 2019 | 2020 |
| --- | --- | --- | --- | --- | --- | --- | --- | --- | --- | --- | --- | --- | --- |
| General median CD4 level | 389 | 355 | 363 | 350 | 364 | 356 | 352 | 335 | 328 | 309 | 301 | 300 | 305 |
| Estimated general median CD4 level | 382 | 374 | 366 | 359 | 352 | 345 | 338 | 331 | 324 | 318 | 312 | 305 | 299 |
| Median CD4 level among homosexual transmission | 419 | 362 | 400 | 362 | 392 | 376 | 369 | 360 | 352 | 330 | 318 | 325 | 322 |
| Estimated median CD4 level among homosexual transmission | 404 | 396 | 389 | 381 | 374 | 366 | 359 | 352 | 345 | 339 | 332 | 326 | 319 |
| Median CD4 level among heterosexual transmission | 382 | 359 | 327 | 319 | 308 | 311 | 307 | 291 | 286 | 282 | 281 | 272 | 281 |
| Estimated median CD4 level among heterosexual transmission | 385 | 353 | 324 | 319 | 313 | 307 | 302 | 297 | 291 | 286 | 281 | 276 | 271 |
| Median CD4 level among IDU | 405 | 427 | 380 | 428 | 421 | 430 | 473 | 454 | 346 | 376 | 321 | 358 | 312 |
| Estimated median CD4 level among IDU | 445 | 435 | 426 | 417 | 409 | 400 | 392 | 383 | 375 | 368 | 360 | 352 | 345 |
| Median CD4 level among others | 308 | 286 | 219 | 279 | 252 | 368 | 311 | 237 | 282 | 267 | 302 | 287 | 252 |
| Estimated median CD4 level among others | 281 | 280 | 280 | 280 | 279 | 279 | 278 | 278 | 278 | 277 | 277 | 277 | 276 |

IDU: Inject drug user.

Supplement table 5. Trends in rate of LP and AHD in Jiangsu, China: Year of change of trend, annual percentage change, and annual average percentage change

| Category | Period | Join-point | APC^a^,95%CI^c^ | P value | AAPC^b^,95%CI^c^ | P value |
| --- | --- | --- | --- | --- | --- | --- |
| Late presentation | 2008-2020 | 0 | 2.7, (2.0, 3.4) | <0.001 | 2.7, (2.0, 3.4) | <0.001 |
| Advanced HIV disease | 2008-2016 | 1 | 1.6, (-2.8, 6.2) | 0.433 | -1.3, (-4.5, 2.0) | 0.435 |
|  | 2016-2020 |  | -6.8, (-13.4, 0.3) | 0.057 |  |  |

^a^ APC, annual percentage change; ^b^ APPC, average annual percent change; ^c^ CI, confidence interval.

Supplement table 6. Trends in rate of LP and AHD among varied transmission route in Jiangsu, China: Year of change of trend, annual percentage change, and annual average percentage change

| Category | Transmission routes | Period | Join-point | APC^a^,95%CI^c^ | P value | AAPC^b^,95%CI^c^ | P value |
| --- | --- | --- | --- | --- | --- | --- | --- |
| Late presentation | Homosexual | 2008-2020 | 0 | 3.2, (2.2, 4.1) | <0.001 | 3.2, (2.2, 4.1) | <0.001 |
|  | Heterosexual | 2008-2020 | 0 | 2.2, (1.5, 2.9) | <0.001 | 2.2, (1.5, 2.9) | <0.001 |
|  | IDU | 2008-2020 | 0 | 7.2, (1.5, 13.2) | 0.017 | 7.2, (1.5, 13.2) | 0.017 |
|  | Others | 2008-2020 | 0 | 1.2, (-0.3, 2.7) | 0.103 | 1.2, (-0.3, 2.7) | 0.103 |
| Advanced HIV disease | Homosexual | 2008-2015 | 1 | 6.0, (-1.2, 13.7) | 0.093 | 0.9, (-3.0, 4.9) | 0.657 |
|  |  | 2015-2020 |  | -5.8, (-10.4, -1.0) | 0.025 |  |  |
|  | Heterosexual | 2008-2015 | 1 | 2.3, (-5.2, 10.4) | 0.509 | -1.4, (-5.7, 3.0) | 0.524 |
|  |  | 2015-2020 |  | -6.4, (-12.2, -0.3) | 0.043 |  |  |
|  | IDU | 2008-2020 | 0 | 5.4, (-0.4, 11.4) | 0.064 | 5.4, (-0.4, 11.4) | 0.064 |
|  | Others | 2008-2020 | 0 | -0.8, (-5.2, 3.8) | 0.705 | -0.8, (-5.2, 3.8) | 0.705 |

LP: Late presentation; AHD: Advanced HIV disease; IDU: Inject drug user; ^a^ APC, annual percentage change; ^b^ APPC, average annual percent change; ^c^ CI, confidence interval.

Supplement table 7. Trend of median CD4 level in Jiangsu, China: Year of change of trend, annual percentage change, and annual average percentage change

| Category | Period | Join-point | APC^a^,95%CI^c^ | P value | AAPC^b^,95%CI^c^ | P value |
| --- | --- | --- | --- | --- | --- | --- |
| Total median CD4 level | 2008-2020 | 0 | -2.0, (-2.5, -1.5) | <0.001 | -2.0, (-2.5, -1.5) | <0.001 |

^a^ APC, annual percentage change; ^b^ APPC, average annual percent change; ^c^ CI, confidence interval.

Supplement table 8. Trends of median CD4 levels among varied transmission route in Jiangsu, China: Year of change of trend, annual percentage change, and annual average percentage change

| Transmission routes | Period | Join-point | APC ^a^,95%CI^c^ | P value | AAPC^b^,95%CI^c^ | P value |
| --- | --- | --- | --- | --- | --- | --- |
| Homosexual | 2008-2020 | 0 | -1.9, (-2.6, -1.3) | <0.001 | -1.9, (-2.6, -1.3) | <0.001 |
| Heterosexual | 2008-2010 | 1 | -8.2, (-13.8, -2.3) | 0.014 | -2.9, (-3.8, -2.0) | <0.001 |
|  | 2010-2020 |  | -1.8, (-2.3, -1.3) | <0.001 |  |  |
| IDU | 2008-2020 | 0 | -1.7, (-4.0, 0.6) | 0.132 | -1.7, (-4.0, 0.6) | 0.132 |
| Others | 2008-2020 | 0 | 0.0, (-1.5, 1.5) | 0.998 | 0.0, (-1.5, 1.5) | 0.998 |

IDU: Inject drug user; ^a^ APC, annual percentage change; ^b^ APPC, average annual percent change; ^c^ CI, confidence interval.
